# Prevalence of insomnia and its impact on daily function amongst Malaysian primary care patients

**DOI:** 10.1186/1447-056X-11-9

**Published:** 2012-11-27

**Authors:** Abu-Hassan Zailinawati, Danielle Mazza, Cheong Lieng Teng

**Affiliations:** 1 Department of Family Medicine, International Medical University, Jalan Rasah, Seremban, Negeri Sembilan, 70300, Malaysia; 2 Department of General Practice, Monash University, Melbourne, Australia

**Keywords:** Insomnia, Psychological morbidity, Prevalence, Primary care, Malaysia

## Abstract

**Background:**

Insomnia is a common public health problem and the prevalence and impact of insomnia in primary care attendees is not well documented in the Asian population.

**Objectives:**

To determine the prevalence of self-reported insomnia symptoms amongst adult primary care attendees and the association with socio-demographic factors; to ascertain the impact of insomnia on daily functioning and to describe the psychological profile of patients with insomnia.

**Methods:**

In this cross-sectional survey, 2049 adult patients (≥18 year old) attending seven primary care clinics in Peninsular Malaysia, completed the questionnaire asking about symptoms of insomnia (defined according to the International Classification of Sleep Disorders and DSM IV criteria) daytime impairment and psychological symptoms (assessed by Hospital Anxiety and Depression Scale).

**Results:**

The response rate was 86.2%. A total of 60% reported insomnia symptoms, 38.9% had frequent insomnia symptoms (>3 times per week), 30.7% had chronic insomnia without daytime consequences and 28.6% had chronic insomnia with daytime dysfunction. Indian ethnicity (OR 1.79; 95%CI, 1.28-2.49), age ≥ 50 or older (OR 1.82; 95%CI, 1.10-3.01), anxiety symptoms (OR 1.65; 95%CI, 1.21-2.22) and depression symptoms (OR 1.65; 95%CI, 1.21-2.26) were risk factors for chronic insomnia with daytime dysfunction. Amongst those with chronic insomnia with daytime dysfunction, 47.8% had anxiety symptoms (OR, 2.01; 95%CI, 1.57-2.59) and 36.5% had depression symptoms (OR, 2.74; 95%CI, 2.04-3.68) based on HADs score. They also had tendency to doze off while driving and to be involved in road traffic accidents.

**Conclusions:**

A third of primary care attendees have insomnia symptoms and chronic insomnia, associated with significant daytime dysfunction and psychological morbidity. By identifying those at risk of having chronic insomnia, appropriate interventions can be commenced.

## Background

Insomnia is a common public health problem with an estimated prevalence in both Western and Asian general populations in the range of 11-50% [1-7]. The wide variation of prevalence is due to differences in definitions of insomnia used, populations studied and research methodology. Studies conducted in Western settings have reported that primary care populations have a higher prevalence of insomnia (64-69%) than the general population [8-10]. Patients in primary care clinics with chronic insomnia have more severe sleep complaints and poorer daytime functioning, and consulted more often [9]. Patients with insomnia have been reported to have twice as many admissions to hospital, more visits to GPs (increased 14%) and undergo more laboratory testing (increased 9%). They were also noted to have clinical consultations an average 12.87 times per year compared to 5.25 per year for the non insomnia patients [8]. The strong association between insomnia and health care utilisation persists even after adjustments for age, gender and chronic disease [10].

Insomnia has a significant impact on individual health particularly those with co-morbid conditions, it has been reported to be associated with chronic diseases such as ischemic heart disease and hypertension, morning headaches and depression [11-13]. Epidemiological studies have demonstrated that lack of sleep is associated with daytime consequences such as poor performance at work [14], impaired memory and concentration [3,10]. Insomnia has also been shown to be related to higher rates of work absenteeism and increased rates of motor vehicle and workplace accidents [15]. All these have significant direct and indirect economic implications [8,10,14-16]. Simon et al. reported that those with insomnia used average 60% higher of total health services compared those without insomnia. Despite the high reported prevalence and public health burden of insomnia worldwide, it remains a problem that is widely under-recognized and under-treated [2,17].

In view of the significant impact on individuals and society, and primary care being the first patients’ encounter, there is a need to explore this issue in primary care settings further. To date, there have been no studies on insomnia in Malaysian or any other Asian primary care settings. This study was conducted in seven primary care clinics in Peninsular Malaysia. We aimed to document the prevalence of self-reported insomnia symptoms and sleep patterns amongst adult primary care attendees in Malaysia, to see if this was associated with socio-demographic and clinical characteristics, to ascertain the impact of insomnia on daily functioning and to describe the psychological profile of patients with insomnia.

## Methods

### Study design

The study was a cross-sectional, self-administered questionnaire survey, conducted in five private primary care clinics and two public primary care clinics in Malaysia. It was conducted between January to November 2007. Primary care providers in Malaysia can be broadly classified into private and government sectors. Since there is no complete sampling frame for the private care providers in Malaysia convenience sampling was employed. Purposive selection of the clinics was based on their varied geographical locations in the northern, central and southern part of Peninsular Malaysia and to ensure representation of the different ethnic groups that make up Malaysia’s multiethnic population. A pilot study was conducted to refine the questionnaire and study protocol [18]. Ethical approval for the study was obtained from the Human Ethics Committees of Monash University, Australia and International Medical University, Malaysia.

### Sampling and enrollment criteria

Due to the large difference in daily number of patients seen in public primary care clinics (200–400 patients per day) and private primary care clinics (20–30 patients per day), one in five patients was invited to participate in the study in the public clinics and consecutive patients were recruited by the doctors in the private clinics. We collected an equal number of patients from each setting.

Participants in the study were aged 18 years and above attending the primary care clinics for any reason. Exclusion criteria were serious illness, impaired memory, those with mental illness, women who were either pregnant or up to 6 weeks postpartum and illiterate patients, as they may not be able to provide accurate responses which may introduce bias to the study.

### Questionnaires

The self-administered questionnaire sought data on socio-demographic factors, sleep, lifestyle issues (cigarette use, caffeine and alcohol consumption) and utilized the Epworth Sleepiness Scale (ESS) [19] to measure daytime dysfunction (sleepiness) and the Hospital Anxiety and Depression Scale (HADs scale) to measure psychological symptoms [20]. The study was conducted in English and Malay as most Malaysians understand either of these languages.

To evaluate their sleep pattern, respondents were asked about difficulty in falling asleep (> 30 min to fall asleep), maintaining sleep (> 3 night-time awakenings), early morning awakening (waking between 2 to 5am) and complaints of un-refreshing sleep (waking non refreshed in the morning) and the frequency and duration of these events.

The presence of any of the above symptoms on at least 3 days in a week was considered to be insomnia, based on the International Classification of Sleep Disorders (ICSD 2001) [21] and DSM IV criteria [22].

Daytime impairment was said to occur when the respondent answered positively to any of the following [23]: trouble functioning during the day due to sleepiness, feeling irritable, feeling anxious, feeling depressed, loss of concentration, exhaustion, decreased work productivity, poor memory, Epworth Sleepiness Scale [19] is ≥ 11.

The respondents with insomnia were classified into 3 categories based on the Italian sleep study [8]:

1) Level 1 - Insomnia (any of the insomnia symptoms on at least 3 days of the week) with absence of daytime dysfunction

2) Level 2 - Chronic insomnia (Level 1 insomnia) with symptoms persisting for > than 4 weeks duration

3) Level 3 - Chronic insomnia (Level 2 insomnia) with daytime impairment.

Psychological symptoms were considered as absent if the HADs score was 0–7, mild if subjects scored 8–10, moderate if 11–14 and severe if 15–21. The scales used in this study have acceptable validity, reliability and test-retest reliability [20,24]. Several studies have shown that HADS is dependable, stable, and consistent in producing the result and has been validated in Malaysia [24-26].

### Statistical analysis

Data were analyzed using the Statistical Package for Social Sciences (SPSS) version 11.0. Statistical comparison of categorical variables and continuous data was undertaken using χ^2^ test and *t*-test respectively. For ordinal variable where linear trend is expected, chi-square test for linear-to-linear association (equivalent to chi-square test for trend) was applied. Statistical significance was set at p< 0.05. In the univariate analysis, insomnia is the dependent variable (this is classified at several levels: insomnia, chronic insomnia, chronic insomnia with daytime consequences). The independent variables are factors such as sex, age group, employment status and lifestyle habits.

## Result

### Demographics and prevalence of insomnia in primary care patients

Of the 2400 questionnaires distributed, 2075 patients returned the survey but 26 incomplete questionnaires were excluded. The response rate varied in different centers from 50.2% to 100%, with an average of 86.2%. The mean age of the respondents was 39 years (SD ± 14.46), and the majority were employed (70.2%) (Table 1).

**Table 1:** Socio-demographic data of participants

**Variable**	**n**	**Percentage (%)**
**Gender**		
Male	908	44.3
Female	1141	55.7
**Age group**		
18-29	694	34.0
30-39	467	22.9
40-49	369	18.1
50-59	301	14.7
> 60	212	10.4
**Ethnic group**		
Malay	1242	60.6
Chinese	401	19.6
Indian	375	18.3
Others	31	1.5
**Marital status**		
Single	548	29.3
Married	1306	65.6
Separated/divorced/widow	102	5.1
**Employment status**		
Employed	1436	70.2
Unemployed	429	21.0
Pensioner	130	6.4
Others	50	2.4
Household income*		
<500	121	6.0
501-1000	479	23.7
1001-2000	720	35.7
2001-3000	333	16.5
3001-4000	156	7.7
4001-5000	70	3.5
>5000	140	6.9
**Educational Level**		
Never attended school	46	2.3
Primary school only	275	13.5
Secondary school only	1076	52.7
Tertiary education	694	31.6

* RM- Ringgit Malaysia, 1 USD = 3.4 RM.

Sixty percent of the study population had at least one of the insomnia symptoms, with 38.9% classified as level 1 insomnia, 30.7% as level 2 insomnia and 28.6% as level 3 insomnia. Chronic (or level 2) insomnia was more prevalent among those aged ≥ 60 years, of Indian ethnicity, those who were separated, divorced or widowed, who earned less than RM 500 per month, who were unemployed or who had a low educational level. There was no significant gender difference in any level of insomnia (Table 2). Amongst all respondents only 6.3% (130) and amongst those with insomnia only 14.2% (113) discussed their sleep problems with their doctors.

**Table 2:** Demographic characteristics of patients with different level of insomnia

	**Level 1 Insomnia n = 798 n (%)**	**Level 2 Insomnia n = 630 n (%)**	**Level 3 Insomnia n = 587 n (%)**
**Gender**			
Male	353 (39.2)	262 (51.2)	243 (47.3)
Female	445 (39.3)	368 (54.9)	344 (51.0)
Chi-square test			χ^2^=1.17, df=1, p>0.05
**Ethnic Group**			
Indian	158 (42.8)*	143 (67.5)*	135 (63.1)*
Non-Indian			(46.4)
Chi-square test			χ^2^=19.5, df=1, p<0.001
**Age Group**			
18-29	260 (37.7)	189 (45.5)	178 (42.6)
30-39	186 (40.3)	137 (47.1)	128 (43.8)
40-49	144 (39.2)	117 (54.9)	108 (50.2)
50-59	124 (41.5)	116 (69.0)	109 (64.9)
>60	80 (38.1)	67 (73.6)*	60 (65.9)*
Chi-square for trend			χ^2^=33.0, df=1, p<0.001
**Marital Status**			
Single	218 (37.7)	158 (44.3)	151 (42.2)
Married	499 (38.6)	406 (55.9)	375 (51.4)
Separated/Divorced/Widow	54 (52.9)*	49 (74.2)*	44 (66.7)*
Chi-square for trend			χ^2^=15.9, df=1, p<0.001
**Household Income**			
<500	62 (52.5)	54 (65.9)*	53 (63.9)*
501-1000	181 (38.1)	145 (57.1)	136 (52.9)
1001-2000	266 (37.2)	212 (52.9)	194 (48.3)
2001-3000	131 (39.8)	102 (51.5)	93 (47.0)
3001-4000	59 (38.1)	45 (46.9)	40 (41.7)
4001-5000	28 (40.0)	19 (44.2)	18 (40.9)
>5000	52 (37.1)	38 (45.8)	38 (45.8)
Chi-square for trend			χ^2^=8.2, df=1, p=0.004
**Employment Status**			
Employed	582 (39.5)	421 (48.3)	390 (44.6)
Unemployed	168 (39.6)	153 (69.9)	146 (66.1)*
Pensioner	44 (33.8)	39 (72.2)*	34 (63.0)
Chi-square test			χ^2^=36.7, df=2, p<0.001
**Educational Level**			
Never attended school	28 (60.9)*	24 (77.4)*	23 (74.2)*
Primary school	125 (46.1)	114 (67.9)	107 (62.6)
Secondary school	386 (36.1)	309 (53.6)	286 (49.4)
Tertiary education	250 (40.4)	182 (44.9)	170 (41.9)
Chi-square for trend			χ^2^=27.7, df=1, p<0.001

* *p*< 0.05 is significant.

Level 1 insomnia – any of the insomnia symptoms occurring more than 3 times per week.

Level 2 insomnia – chronic insomnia (any of the insomnia symptoms occurring more than 3 times per week, persist more than 4 weeks).

Level 3 insomnia – chronic insomnia with daytime consequences.

Private clinics attendees tended to have more insomnia than patients attending public clinics (47.6% vs. 30.5%, p<0.001). However, there was significantly more chronic insomnia in public compared to private clinics (61.9% vs. 47.9%, p<0.001) and more chronic insomnia with daytime dysfunction in public clinics (55.3% vs. 45.8%, p<0.001).

### Pattern of insomnia in primary care patients

Among those who reported insomnia, difficulty initiating sleep and un-refreshed sleep were noted in 32.6% and 36.4% respectively, while difficulty maintaining sleep (20.5%) and early morning awakening (14.8%) were less frequent. A total of 57.4% and 55.3% of those who had chronic insomnia reported having difficulty initiating sleep and un-refreshed sleep respectively. While 32.5% of those with chronic insomnia had difficulty maintaining sleep and 24.5% had early morning awakening. The respondents who were older than 60 years old with chronic insomnia experienced more difficulty in initiating sleep, compared to those between 18 to 29 years old (47.2% vs. 71.7%, p<0.001). The younger age group reported more of un-refreshed sleep compared to the elderly (68.5% vs. 44.1%, p<0.001).

Those who had insomnia and chronic insomnia took an average of 45 min (SD ± 41.5), and those who did not have insomnia took an average of 17.7 min, (SD ± 14.6) to fall asleep. Chronic insomniacs range of sleep latency (duration of time from “lights out,” or bedtime, to the onset of sleep) was from 1 min to 5 h. Those who had insomnia, slept significantly less (about 5.7 h per night) (SD ± 1.25), compared to those without insomnia about 6.6 h per night (SD ± 1.00). About 14.5% of those with insomnia slept less than 4 h per night, and a total of 39.6% slept less than 5 h per night.

### Lifestyle factors associated with insomnia pattern in primary care patients

Table 3 shows the association between lifestyle factors and the different levels of insomnia. Smokers were more likely to have un-refreshed sleep, (49.5% vs. 32.9%, p<0.001). Those who drank more than 5 cups of caffeinated drinks per day, tended to have more symptoms of insomnia with more difficulty falling asleep, (68.6% vs. 31.4%, p<0.001) and more un-refreshed sleep (77.1% vs. 22.9%, p<0.001).

**Table 3:** Lifestyle of the respondents (non insomnia and the different level of insomnia)

	**Non-insomniacs n=819 n (%)**	**Level 1 insomnia n=798 n (%)**	**Level 2 insomnia n=630 n (%)**	**Level 3 insomnia n=587 n (%)**
Cigarette smoking	73 (17.6)	208 (26.1)*	153 (24.3)*	145 (24.7)*
Consume caffeinated drinks	304 (73.3)	605 (76.0)	483 (76.9)	454 (77.5)
Number of cups caffeinated drinks per day				
1-2	248 (81.0)	428 (70.7)	343 (71.0)	318 (69.6)
3-5	56 (18.3)	143 (23.6)	112 (23.3)	110 (24.2)
>5	2 (0.7)	34 (5.6)*	28 (5.8)*	27 (5.9)*
Consume coffee 1 h before Bedtime	28 (7.1)	93 (12.4)*	77 (13.0)*	74 (13.4)*
Alcohol	18 (4.4)	45 (5.7)	38 (6.1)	34 (5.8)
Sedative use	5 (1.2)	67 (8.4)*	57 (9.1)*	56 (9.5)*

* p<0.05 is significant.

Level 1 insomnia – any of the insomnia symptoms occurring more than 3 times per week.

Level 2 insomnia – chronic insomnia (any of the insomnia symptoms occurring more than 3 times per week, persist more than 4 weeks).

Level 3 insomnia – chronic insomnia with daytime consequences.

While only 3.9% of respondents consumed alcohol in this study there were no differences in sleep patterns between those who drank and those who did not.

Respondents with insomnia take more sedatives (8.4% vs. 1.2% p<0.001). The ‘sleep medication’ that they presumed helped them included paracetamol, antihistamines, anti-depressants, anxiolytics and benzodiazepines (Table 3).

### The impact of insomnia on daily function amongst primary care patients

The majority of those who had any level of insomnia (83.3%) reported fair to poor quality of sleep. Those with insomnia symptoms were more likely to report significant daytime sleepiness (66.2% vs. 52.2%, p<0.001) and this was supported by a significantly higher percentage of them having an ESS score ≥11 (28.2% vs. 18.3%, p<0.001).

Those with insomnia were more likely to exhibit psychological symptoms such as, easily irritable (50.8% vs. 35.4%, p<0.001), anxious (57.5% vs. 36.0%, p<0.001) and felt depressed (45.1% vs. 25.4%, p<0.001). They also reported loss of concentration at work (37.9% vs. 21.8%, p<0.001), they felt tired easily (72.1% vs. 60.7%, p<0.001 and had poorer memory (43.8% vs. 29.8%, p<0.001). They perceived that they were less productive (37.4% vs. 18.9%, p<0.001) and felt that they poorer health (8.1% vs. 2.2%, p<0.001).

Those with chronic insomnia were more likely to report driving while sleepy (42.5% vs. 35.5%, p=0.032, OR 1.12, 95%CI 1.01-1.25) and about one in five reported to dozing off while driving (19.1% vs. 13.1%, p=0.016, OR 1.07, 95%CI 1.01-1.14). Four percent of those chronic insomniacs were involved in road traffic accidents due to sleepiness (3.9% vs. 1.1%, p=0.007, OR 1.03, 95%CI 1.00-1.05).

### The psychological profile of patients with insomnia

Of the 780 primary care attendees that had insomnia, a total of 47.8% (285) had anxiety symptoms based on HADS, with OR 2.01; 95%CI 1.57-2.59 (60.0% were categorised as having mild symptoms, 29.1% moderate and 2.9% severe anxiety symptoms). A total of 36.5% (285) were considered to have depression (OR 2.74; 95%CI 2.04-3.68), with 70.5% of them categorised as mild, 27.4% moderate and 2.1% had severe symptoms of depression. Further analysis in the different category of insomnia, either chronic insomnia or those with daytime dysfunction showed that the percentage who were positive for both anxiety and depression were almost the same. Interestingly, of those with anxiety, 74.4% experienced insomnia, as did 80.1% of those with depression.

### Multivariate analyses

Logistic regression analysis indicated that the likelihood of someone having chronic insomnia with daytime consequences was independently associated with being Indian ethnicity, (OR 1.79; 95%CI 1.28-2.49), aged 50 or older, (OR 1.82; 95%CI 1.10-3.01), having anxiety symptoms, (OR 1.65; 95%CI 1.21-2.22) and depression symptoms, (OR 1.65; 95%CI 1.21-2.26).

## Discussion

The study population was representative of the primary care attendees in the selected primary care clinics in the Peninsular Malaysia. In our study, almost half of the patients attended the clinics of any reasons, experienced at least one of the insomnia symptoms, and one third experienced chronic insomnia. This confirms that the prevalence of insomnia in primary care is higher compared to the general population in Malaysia [27,28] and other Asian [4-7] and Western countries [1-3,17] using the same definition. In Malaysia, 33.8% of the general population were reported to have insomnia symptoms and 12.2% had chronic insomnia [27]. The high prevalence is probably due to the possible underlying physical and mental health problem that brought them to the primary care clinics. Primary care attendees presented with a variety of reasons for visits. Various studies suggest that majority of those with insomnia seen in primary care clinics have co-morbid conditions [12,13]. Hence the new phrase of “comorbid insomnia’ emerged from the 2005 National Institutes of Health’s (NIH) conference [13]. Comorbid insomnia refers to insomnia related to certain medical conditions (psychiatric or medical disorders), medicines, and certain substances such caffeine. Unfortunately, the reason for visits and co-morbidities for the sample population of this study is out of the scope of this paper.

This study shows that chronic insomnia is highly prevalent (30.7%) if compared to most studies in general population [2-7] which generally reported 9-15%. This finding indicates that chronic insomnia is common among those seeking health care; perhaps because associated co-morbidities such as medical or psychiatric disorders cause symptoms of insomnia. The importance of chronic insomnia is the association with poor daytime functioning which may in turn affect work efficiency and productivity and may be detrimental if their working environment demands high cognitive skills and constant alertness.

Our finding that chronic insomnia is significantly associated with daytime sleepiness and falling asleep whilst driving may lead to a higher risk of road traffic accidents, concurs with other studies [15,29] and is of serious public health concern. Road traffic accidents constitute 5.7% of the total burden of disease in Malaysia, which is the top three, after ischaemic heart disease (9.8%) and cardiovascular disease (6.4%) [30]. Malaysian government is addressing these issues actively to reduce road traffic accidents [31]. However, there is a need to include widespread national campaigns to advocate enough sleep, create awareness of the symptoms and effect of sleep deprivation and promote help seeking in those suffering from insomnia. Besides insomnia and daytime sleepiness, reports of personality change in subjects with insomnia and associated cognitive changes could also contribute to traffic accidents [32], however, there are many other causes of daytime sleepiness which were not explored in this study [23].

Chronic insomnia may be a symptom of a psychiatric disorder or predispose or be a risk factor for these conditions [12,33-35]. This study confirmed the association between chronic insomnia and symptoms of depression and anxiety. We also noted that there was no particular pattern of insomnia associated with depression supporting others who have noted that the classical ‘early morning wakening’ symptom is not necessarily linked to depression [12]. We believe therefore that where a patient is found to suffer from any form of insomnia, be that difficulty falling asleep or maintaining sleep, early wakening or waking up un-refreshed further exploration of psychological symptoms should be undertaken. Some authors even suggest that treatment of insomnia may reduce the risk of psychological disorders [12,34].

Our findings related to the socio-demographic variables associated with insomnia concur with the results of other studies [1-7,17]. The high prevalence of chronic insomnia amongst the elderly probably due to the progressive inactivity, dissatisfaction with social life and concurrent medical and psychiatric problems [2,10,17]. The lack of gender predilection for insomnia in Malaysian women further substantiates the result of a meta-analysis [36] and confirms result of the other local studies [27,28] which report that Asian countries have less gender (female) predisposition to insomnia than Western countries. In a sub-analysis (not shown) the Indian ethnicity were significantly unemployed, had low educational status and reported more depressive symptoms based on HADs scoring; which may explain the association with the increase risk of having insomnia symptoms compared to other ethnic groups.

Like other studies [1,9], not many of the patients with insomnia consulted a physician for sleep problems. Despite the high prevalence of symptoms, less than ten percent of those with insomnia were on any type of sedatives. Unfortunately, we did not know type of sedatives used by the patients. This study is an important study in primary care population in both private and public clinics in Malaysia as it shows that insomnia is common, under-recognised and under-treated. We were however unable to establish a causal link between insomnia and psychiatric disorders due to the cross-sectional design and the lack of clinical interview for diagnosing disorders in this study. A longitudinal study is needed to understand the relationship between sleep problems and mental disorders. Future research should also explore the types of interventions which might be successful in the treatment and prevention of insomnia.

There are several limitations of this study. The study populations in this study were from the selected clinics in the west coast of Peninsular Malaysia, thus result may not be extrapolated to the all primary care clinics in Malaysia. The cross-sectional study design was only able to show the association between patients with insomnia symptoms and the daytime-dysfunctions and psychological morbidity. This design is unable to establish the causal link. Furthermore, insomnia is defined as subjective complaints by the respondent; therefore there may be some elements of recall and self-report bias.

## Conclusion

Our findings confirm that the presence of insomnia symptoms in the primary care population is associated with a higher risk of impaired functioning, poor mental health and poor well being. By identifying those at risk of having chronic insomnia, appropriate intervention can be commenced. There is a need to acquire a better understanding of sleep problems and be aware that patients do not always discuss their sleep difficulties with health care providers. Educational program are needed to promote the awareness of sleep problems for undergraduates, post graduates and practicing physicians.

## Competing interests

The authors declare that they have no competing interests.

## Authors’ contributions

ZAH participated in the proposal, design, coordinating, carried out of the study and drafted the manuscript. DM participated in the proposal, design, coordinating the study, and drafted the manuscript. CLT participated in the proposal, design, coordinating the study, performed the statistical analysis and drafted the manuscript. All authors read and approved of the final manuscript.

## References

[B1] Ustun TB Privett M Lecrubier Y Form, frequency and burden of sleep problem in general health care: a report from the WHO collaborative study on psychological problem in general health care Eur Psychiatry 1996 11 Supp 1 5S 10S

[B2] Ohayon MM Epidemiology of insomnia: what we know and what we still need to learn Sleep Med Rev 2002 6 2 97 111 10.1053/smrv.2002.0186 12531146

[B3] Groeger JA Zijlstra FR Dijk DJ Sleep quantity, sleep difficulties and their perceived consequences in a representative sample of some 2000 British adults J Sleep Res 2004 13 4 359 371 10.1111/j.1365-2869.2004.00418.x 15560771

[B4] Kim K Uchiyama M Okawa M Liu X Ogihara R An epidemiological study of insomnia among the Japanese general population Sleep 2000 23 1 41 47 10678464

[B5] Yeo BK Perera IS Kok LP Tsoi WF Insomnia in the community Singapore Med J 1996 37 282 284 8942230

[B6] Li RH Wing YK Ho SC Fong S Gender differences in insomnia: a study in the Hong Kong Chinese population J Psychosom Res 2002 53 601 609 10.1016/S0022-3999(02)00437-3 12127178

[B7] Ohayon MM Hong SC Prevalence of insomnia and associated factors in South Korea J Psychosom Res 2002 53 593 600 10.1016/S0022-3999(02)00449-X 12127177

[B8] Terzano MG Parrino L Cirignotta F Ferini-Strambi L Gigli G Rudelli G Studio Morfeo: insomnia in primary care, a survey conducted on the Italian population Sleep Med 2004 5 67 75 10.1016/j.sleep.2003.09.006 14725829

[B9] Aikens JE Rouse ME Help-seeking for insomnia among adult patients in primary care J Am Board Fam Pract 2005 18 4 257 260 10.3122/jabfm.18.4.257 15994471

[B10] Simon GE VonKorff M Prevalence, burden, and treatment of insomnia in primary care Am J Psychiatry 1997 154 1417 1423 9326825 10.1176/ajp.154.10.1417

[B11] Leineweber C Kecklund G Janszky I Akerstedt T Orth-Gomer K Poor sleep increases the prospective risk for recurrent events in middle-aged women with coronary disease. The stockholm female coronary risk study J Psychosom Res 2003 54 2 121 127 10.1016/S0022-3999(02)00475-0 12573733

[B12] Ford DE Kamerow DB Epidemiologic study of sleep disturbances and psychiatric disorders: an opportunity for prevention JAMA 1989 262 1479 1484 10.1001/jama.1989.03430110069030 2769898

[B13] Roth T Comorbid insomnia: current directions and future challenges Am J Manag Care 2009 15 Suppl S6 S13 19298104

[B14] Léger D Guilleminault C Bader G Lévy E Paillard M Medical and socio-professional impact of insomnia Sleep 2002 25 6 625 629 12224841

[B15] Connor J Norton R Ameratunga S Robinson R Civil I Dunn R Driver sleepiness and risk of serious injury to car occupants: population based case control study BMJ 2002 324 1125 1129 10.1136/bmj.324.7346.1125 12003884 PMC107904

[B16] Ozminkowski RJ Wang S Walsh JK The direct and indirect costs of untreated insomnia in adults in the United States Sleep 2007 30 3 263 273 17425222 10.1093/sleep/30.3.263

[B17] Sateia MJ Doghramji K Hauri PJ Morin CM Evaluation of chronic insomnia. An american academy of sleep medicine review Sleep 2001 23 243 308 10737342

[B18] Zailinawati AH Schattner P Mazza D Doing a pilot study: why is it essential? Mal Fam Physician 2006 1 2&3 70 73 PMC4453116 27570591

[B19] John MA A new method of measuring daytime sleepiness: the Epworth Sleepiness Scale Sleep 1991 14 54 55 10.1093/sleep/14.6.540 1798888

[B20] Zigmond AS Snaith RP The hospital anxiety and depression scale Acta Psychiatr Scand 1983 67 361 370 10.1111/j.1600-0447.1983.tb09716.x 6880820

[B21] International Classification of Sleep Disorders, Revised: Diagnostic and Coding Manual 2001 American Sleep Disorders Association

[B22] Diagnostic and Statistical Manual of Mental Disorders 1994 4th American Psychiatric Association

[B23] Edinger JD Bonnet MH Bootzin RR American Academy of Sleep Medicine Work Group Derivation of research diagnostic criteria for insomnia: report of an American Academy of Sleep Medicine Work Group Sleep 2004 27 8 1567 1596 15683149 10.1093/sleep/27.8.1567

[B24] Herrmann C International experiences with the Hospital anxiety and depression scale- A review of validation data and clinical results J Psychosom Res 1997 42 1 17 41 10.1016/S0022-3999(96)00216-4 9055211

[B25] Lam CL Pan PC Chan AW Can the hospital anxiety and depression (HAD) scale be used on Chinese elderly in general practice? Fam Pract 1995 12 149 154 10.1093/fampra/12.2.149 7589936

[B26] Quek KF Atiya AS Heng NC Beng CC Validation of the hospital anxiety and depression scale and the psychological disorder among premature ejaculation subjects Int J Impot Res 2007 19 321 325 10.1038/sj.ijir.3901528 17136103

[B27] Zailinawati AH Ariff K Nurjahan M Teng CL Epidemiology of insomnia in Malaysian adults: a community-based survey in 4 urban areas Asia Pac J Public Health 2008 20 3 224 233 10.1177/1010539508316975 19124316

[B28] Kamil MA Teng CL Hassan SA Snoring and breathing pauses during sleep in the Malaysian population Respirology 2007 12 3 375 380 10.1111/j.1440-1843.2007.01030.x 17539841

[B29] Nor’Aishah AB Rampal KG Study of fatigue amongst bus drivers 2004, National University of Malaysia: Masters Thesis in Public health (Occupational Health)

[B30] Malaysia; health situation and trend 2010 [cited 2011 29th September]; Available from: http://www.wpro.who.int/countries/maa/2010/health_situation.htm

[B31] Malaysian Institute of Road Safety Research (MIROS) 2010 [cited 2011 29th September]; Available from: http://www.miros.gov.my/web/guest/national

[B32] Drowsy driving and automobile crashes 1998 National Center on Sleep Disorders Research and National Highway Traffic Safety Administration

[B33] Hohagen F Rink K Käppler C Prevalence and treatment of insomnia in general practice: a longitudinal study Eur Arch Psychiatry Clin Neurosci 1993 242 329 336 10.1007/BF02190245 8323982

[B34] Fong SY Wing YK Longitudinal follow up of primary insomnia patients in a psychiatric clinic Aust N Z J Psychiatry 2007 41 611 617 10.1080/00048670701400016 17558624

[B35] Vollrath M Wicki W Angst J The Zurich study VIII. Insomnia: association with depression, anxiety, somatic syndromes, and course of insomnia Eur Arch Psychiatry Neurol Sci 1989 239 2 113 124 10.1007/BF01759584 2806334

[B36] Zhang B Wing YK Sex differences in insomnia: a meta-analysis Sleep 2006 29 85 93 16453985 10.1093/sleep/29.1.85

